# Hyperandrogenism-induced Athletic Excellence in a Young Patient with Adrenocortical Carcinoma: 10 Years of Remission

**DOI:** 10.1210/jcemcr/luaf170

**Published:** 2025-07-31

**Authors:** Takashi Kono, Satomi Kono, Yasuhiro Nakamura, Yoichi Fujii, Hironobu Sasano, Tomoaki Tanaka

**Affiliations:** Department of Molecular Diagnosis, Chiba University Graduate School of Medicine, Chiba 260-8670, Japan; Research Institute of Disaster Medicine, Chiba University, Chiba 260-8670, Japan; Department of Molecular Diagnosis, Chiba University Graduate School of Medicine, Chiba 260-8670, Japan; Division of Pathology, Faculty of Medicine, Tohoku Medical and Pharmaceutical University, Miyagi 983-8536, Japan; Department of Pathology and Tumor Biology, Graduate School of Medicine, Kyoto University, Kyoto 606-8501, Japan; Department of Molecular Diagnosis, Chiba University Graduate School of Medicine, Chiba 260-8670, Japan; Department of Pathology, Tohoku University School of Medicine, Sendai, Miyagi 980-8575, Japan; Department of Molecular Diagnosis, Chiba University Graduate School of Medicine, Chiba 260-8670, Japan; Research Institute of Disaster Medicine, Chiba University, Chiba 260-8670, Japan

**Keywords:** adrenocortical carcinoma, hyperandrogenism, athletics, androgen, virilization

## Abstract

A 17-year-old female softball player presented with progressive virilization and markedly enhanced athletic performance. Laboratory evaluation revealed elevated serum testosterone with autonomous adrenal secretion. Imaging analysis demonstrated an 8-cm right adrenal mass. Adrenalectomy was performed, and histopathological examination confirmed stage II adrenocortical carcinoma (ACC) with a Ki-67 labeling index of 14%. Postoperatively, she received adjuvant mitotane therapy. At 34 months, a solitary hepatic metastasis was successfully treated with radiofrequency ablation and combination chemotherapy. She has remained disease-free for more than 8 years following initial surgery. This case demonstrates the clinical impact of pathological hyperandrogenism on athletic performance and highlights the potential for long-term remission in young patients with aggressive ACC managed through multimodal therapy.

## Introduction

Adrenocortical carcinoma (ACC) is a rare endocrine malignancy with an incidence of 1 to 2 cases per million [1]. Hormonal excess is the predominant presenting feature, with approximately 20% exhibiting isolated androgen excess and 22% combined cortisol-androgen secretion [2]. Androgen excess in females typically manifests as virilization—hirsutism, voice deepening, and menstrual irregularities.

Excess androgens enhance skeletal-muscle development and athletic performance through multiple mechanisms: increased protein synthesis, preferential type II muscle fiber development, and improved neuromuscular adaptation [3], resulting in measurable performance gains [4]. While androgenic effects have been well studied therapeutically, pathological hyperandrogenism from endocrine malignancies remains poorly understood in sports medicine, particularly during adolescence [5].

This report describes ACC-induced hyperandrogenism in a 17-year-old athlete, quantifying exceptional performance gains through standardized assessments and highlighting the anabolic impact of pathological androgens.

## Case Presentation

A 17-year-old female varsity softball player presented with a 2-year history of progressive hirsutism, amenorrhea, and voice deepening. Following a national training program, her performance metrics (grip strength, sprint time) exceeded age-matched norms by >2 SD. Her performance gains exceeded her teammates, without exogenous steroid use.

**Figure 1.**
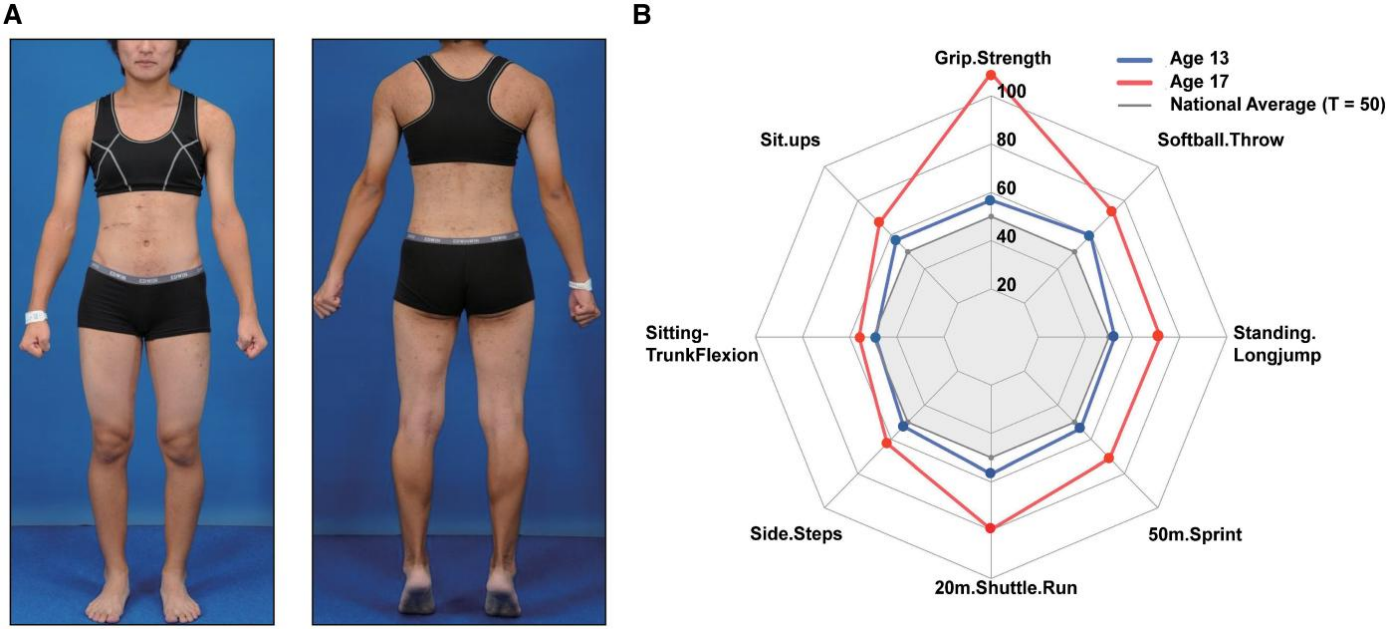
Clinical presentation and athletic performance enhancement. (A) Preoperative photographs showing virilization signs with increased muscle mass and male-pattern body habitus. (B) Radar chart comparing standardized physical fitness measurements (T-scores) at ages 13 (solid blue line) and 17 years (solid red line) relative to age-matched national norms (solid gray reference line, T = 50). Marked improvements in grip strength, sprint speed, and other performance metrics correlate with the period of pathological hyperandrogenism.

Menarche occurred at age 12 with regular cycles until age 15, when oligomenorrhea developed alongside worsening hirsutism. Physical examination findings are shown in Fig. 1A.

Physical fitness assessments (T-scores: mean 50, SD 10) revealed improvements from ages 13 to 17, particularly grip strength (T 56.8 → 109.3), nearly doubling Japanese female norms [6]. Similar gains occurred in cardiorespiratory endurance [7], power output, and muscular strength (Table 1, Fig. 1B).

**Table 1.** Physical fitness test results and T-scores in a female patient with androgen-producing adrenocortical carcinoma from ages 13 to 17 years

Parameter	Age 13		Age 16		Age 17	
	Raw	T-score	Raw	T-score	Raw	T-score
Muscle function						
Grip strength	27 kg	56.8	46 kg	88.0	57 kg	109.3
Power output						
Standing long jump	175 cm	52.3	200 cm	62.3	225 cm	71.7
Softball throw	18 m	49.7	25 m	73.2	25 m	73.2
Speed						
50 m sprint	8.5 second	53.7	7.29 second	67.2	6.88 second	71.1
Endurance						
20 m shuttle run	73 counts	56.7	101 counts	73.9	116 counts	79.7
Sit-ups	27 counts	56.9	28 counts	57.8	35 counts	67.0
Trunk mobility						
Sitting trunk flexion	43 cm	49.1	42 cm	45.5	54 cm	56.0
Side steps	47 counts	52.2	50 counts	50.6	55 counts	52.4

T-score = 50 + 10 × [(raw value − population mean)/population SD]. T-scores are standardized to a population mean of 50 and SD of 10 for age- and sex-matched Japanese adolescents. Normative values were derived from the National Physical Fitness Survey (2008-2012; n = 12 405 females) conducted by the Japan Sports Agency.

Adrenocortical carcinoma diagnosed at age 17.

Physical examination revealed height 165.7 cm, weight 62.4 kg, body mass index 22.7 kg/m², and blood pressure 130/70 mmHg. Virilization signs included hirsutism (Ferriman-Gallwey score 14; normal <8), acne, clitoromegaly (1 cm), and increased muscle mass without cushingoid features.

## Diagnostic Assessment

Endocrine evaluation revealed androgen elevation (Table 2): testosterone 65.2 ng/mL (226 nmol/L) [reference 0.11-0.47 ng/mL (0.38-1.63 nmol/L)], dehydroepiandrosterone sulfate (DHEA-S) 1970µg/dL (53.8 µmol/L) [reference 51-321 µg/dL (1.39-8.76 µmol/L)], and androstenedione 55 ng/mL (192 nmol/L) [reference 0.11-0.39 ng/mL (0.38-1.36 nmol/L)]. High-dose dexamethasone suppression testing demonstrated inadequate cortisol suppression (9.4 µg/dL (259 nmol/L) after 8 mg [normal <1.8 µg/dL (<50 nmol/L)] with a paradoxical increase in testosterone, confirming autonomous hormone production.

**Table 2.** Baseline hormonal assessment and loading test results

Parameter	Value(SI units)	Reference range(SI units)
ACTH—08:00	7.7 pg/mL (1.7 pmol/L)	7.2-63.3 pg/mL (1.6-13.9 pmol/L)
ACTH—20:00	<5.0 pg/mL (<1.1 pmol/L)	<10 pg/mL (<2.2 pmol/L)
Cortisol—08:00	7.0 µg/dL (193 nmol/L)	6.2-19.4 µg/dL (171-535 nmol/L)
Cortisol—24:00	4.0 µg/dL (110 nmol/L)	<10 µg/dL (<276 nmol/L)
DHEA-S	1970 µg/dL (53.8 µmol/L)	51-321 µg/dL (1.39-8.76 µmol/L)
E2	72 pg/mL (264 pmol/L)	30-400 pg/mL (110-1468 pmol/L)
Testosterone	65.2 ng/mL (226 nmol/L)	0.11-0.47 ng/mL (0.38-1.63 nmol/L)
Androstenedione	55 ng/mL (192 nmol/L)	0.11-0.39 ng/mL (0.38-1.36 nmol/L)
TSH	0.903 µIU/mL (0.903 mIU/L)	0.4-4.5 µIU/mL (0.4-4.5 mIU/L)
FT3	2.59 pg/mL (3.98 pmol/L)	2.3-4.2 pg/mL (3.5-6.5 pmol/L)
FT4	0.99 ng/dL (12.7 pmol/L)	0.8-1.8 ng/dL (10.3-23.2 pmol/L)
LH	0.05 mIU/mL (0.05 IU/L)	0.5-7.63 mIU/mL (0.5-7.63 IU/L)
FSH	0.06 mIU/mL (0.06 IU/L)	1.5-3.34 mIU/mL (1.5-3.34 IU/L)
Urine cortisol (24 hours)	86.6 µg/day (239 nmol/day)	10-100 µg/day (27.6-276 nmol/day)

Parameter	Baseline(SI units)	After 2 mg(SI units)	After 8 mg(SI units)	Reference range(SI units)
ACTH	7.7 pg/mL (1.7 pmol/L)	<1.0 pg/mL (<0.22 pmol/L)	<1.0 pg/mL (<0.22 pmol/L)	7.2-63.3 pg/mL (1.6-13.9 pmol/L)
Cortisol	7.0 µg/dL (193 nmol/L)	8.6 µg/dL (237 nmol/L)	9.4 µg/dL (259 nmol/L)	< 1.8 µg/dL (< 50 nmol/L)
Androstenedione	55 ng/mL (192 nmol/L)	ND	87 ng/mL (304 nmol/L)	0.11-0.39 ng/mL (0.38-1.36 nmol/L)
DHEA-S	1970 µg/dL (53.8 µmol/L)	ND	2640 µg/dL (71.9 µmol/L)	51-321 µg/dL (1.39-8.76 µmol/L)
Testosterone	65.2 ng/mL (226 nmol/L)	94.7 ng/mL (328 nmol/L)	92.9 ng/mL (322 nmol/L)	0.11-0.47 ng/mL (0.38-1.63 nmol/L)

Dexamethasone suppression test results

Abbreviations: DHEA-S, dehydroepiandrosterone sulfate; E2, estradiol; ND, not detected; FT3, free T3; FT4, free T4.

**Figure 2.**
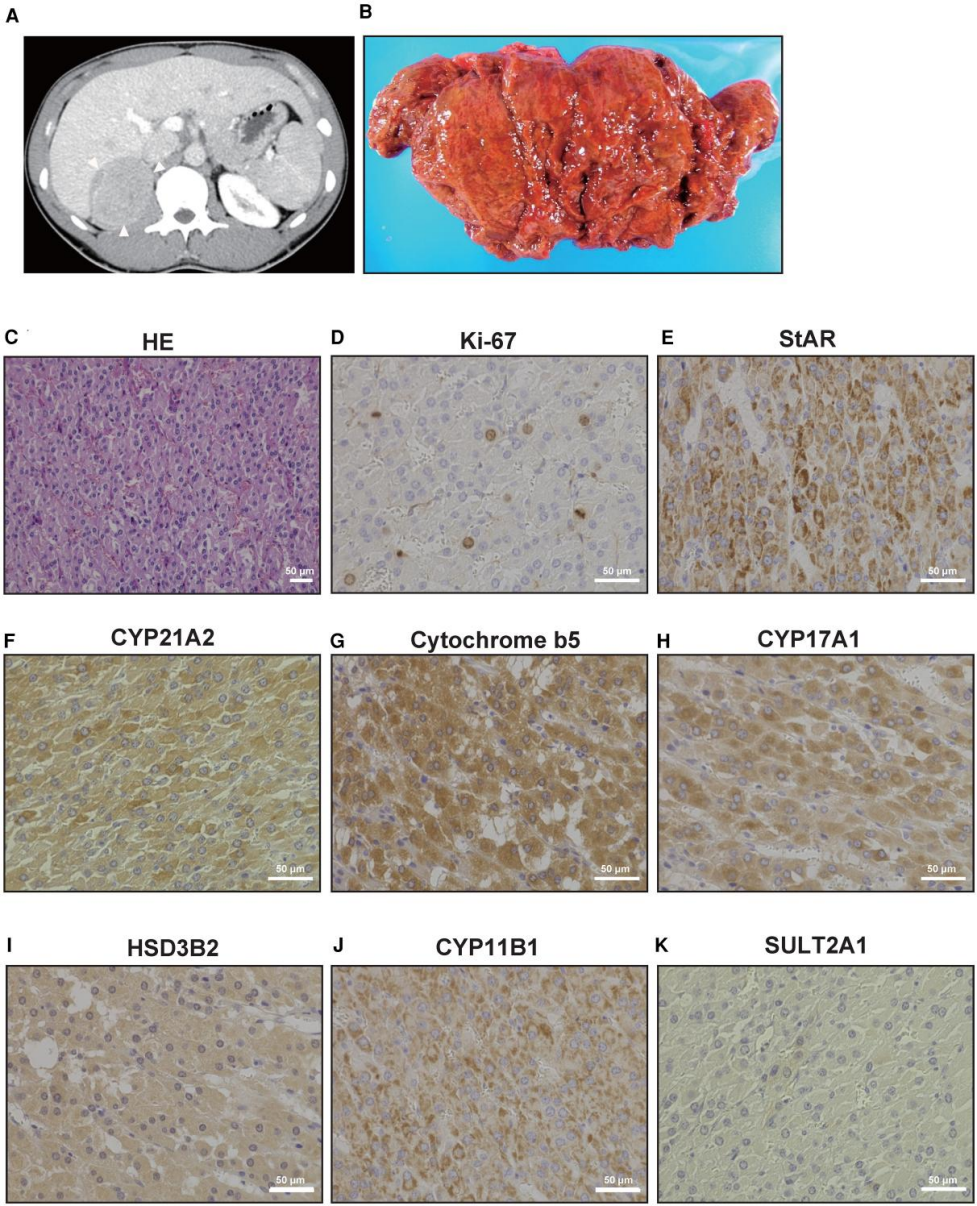
Radiological, pathological, and immunohistochemical characteristics of the virilizing adrenocortical carcinoma. (A) Contrast-enhanced computed tomography showing a large heterogeneous mass in the right adrenal gland with areas of necrosis and hemorrhage. (B) Gross appearance of the resected tumor demonstrating a reddish-brown, well-encapsulated mass with areas of hemorrhage and necrosis. (C) Hematoxylin and eosin staining showing typical adrenocortical carcinoma morphology with sheets of polygonal cells, nuclear pleomorphism, and increased mitotic activity (×200 magnification). (D) Ki-67 immunostaining revealing high proliferative activity with scattered positive nuclei (×400 magnification). (E) StAR protein immunostaining showing moderate cytoplasmic positivity (++), confirming steroidogenic capacity (×400 magnification). (F) CYP21A2 (21β-hydroxylase) immunostaining demonstrating weak to moderate positivity (+/++), consistent with relatively suppressed cortisol biosynthesis (×400 magnification). (G) Cytochrome *b*_5_ immunostaining showing intense diffuse positivity (+++), indicating enhanced 17,20-lyase activity promoting androgen production (×400 magnification). (H) CYP17A1 (17α-hydroxylase/17,20-lyase) immunostaining revealing strong diffuse positivity (+++), explaining the robust androgen biosynthetic capacity (×400 magnification). (I) HSD3B2 (3β-hydroxysteroid dehydrogenase) immunostaining showing strong positivity (+++), confirming active steroidogenesis (×400 magnification). (J) CYP11B1 (11β-hydroxylase) immunostaining demonstrating moderate positivity (++), indicating preserved late-stage cortisol biosynthetic capacity (×400 magnification). (K) SULT2A1 (sulfotransferase 2A1) immunostaining showing minimal expression (±), suggesting that elevated dehydroepiandrosterone sulfate levels were due to substrate excess rather than enhanced enzyme activity (×400 magnification). The immunohistochemical profile explains the selective androgen hypersecretion: strong CYP17A1 and cytochrome *b*_5_ expression drives preferential 17,20-lyase activity, while relatively weak CYP21A2 expression limits cortisol production, collectively resulting in extreme testosterone elevation (139-fold above normal) with maintained cortisol levels. Scale bars represent 50 μm in all panels.

Computed tomography (CT) revealed an 8-cm heterogeneous right adrenal mass with contrast enhancement (Fig. 2A), without metastases. Endocrine and imaging findings established the diagnosis of androgen-producing ACC with subclinical Cushing syndrome.

## Treatment

The patient underwent laparoscopic adrenalectomy at age 17. R0-resection was achieved, with an intact capsule and negative margins, and discharge was on postoperative day 3.

Pathology revealed a 13.5 × 8.5 × 8.3 cm tumor weighing 119 g with high-grade nuclear atypia, >5 mitoses per 50 high-power fields and Ki-67 14%. Six of 9 Weiss criteria—high nuclear grade, elevated mitotic rate, atypical mitoses, eosinophilic cytoplasm, diffuse architecture, and capsular invasion—established high-grade ACC (Fig. 2C and 2D). The final stage was T2N0M0 (stage II).

Postoperatively, mitotane was escalated from 1.5 to 4.5 g/day over 3 weeks, maintaining plasma 14 to 20 mg/L by regular monitoring [8]. Hydrocortisone 40 mg daily prevented adrenal insufficiency, and this regimen accords with current guidelines for high-risk ACC [9-12].

## Outcome and Follow-up

Following confirmation of high-grade ACC (stage II; Ki-67 14%), we performed molecular and immunohistochemical studies to elucidate the extreme androgen excess.

Comprehensive immunohistochemistry of the resected tumor demonstrated intense staining for CYP17A1 and cytochrome *b*_5_, indicating 17,20-lyase activity that preferentially channels precursors toward androgen synthesis. HSD3B2 was likewise strongly positive, whereas CYP21A2 showed only a weak to moderate signal, explaining relatively preserved cortisol production despite massive androgen output. StAR and CYP11B1 were moderately expressed; SULT2A1 was minimal, confirming selective androgen hypersecretion (Fig. 2E-2K).

**Figure 3.**
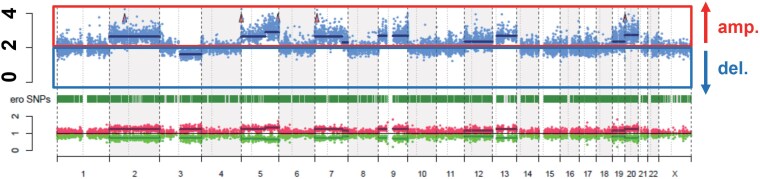
Genomic characterization of the tumor. Copy number variation profile demonstrating characteristic chromosomal alterations, including amplifications (solid red box, amp.) in chromosomes 1q, 2p, and 5p and deletions (solid blue box, del.) in chromosomes 3, 11, and 22, consistent with the genomic landscape typically observed in adrenocortical carcinoma.

Whole-exome sequencing identified somatic *BCOR*-P1384R and *HDAC9*-R947P mutations, reported in <3% of ACCs and predicted pathogenic by in silico analysis [13, 14]. *TP53* and *CTNNB1*, the most common drivers in adult ACC, were wild-type. Genome-wide copy-number profiling revealed the gains and losses typical of ACC (Fig. 3) [15-17], fitting a rare *BCOR/HDAC9*-mutant subset of the recognized spectrum.

**Figure 4.**
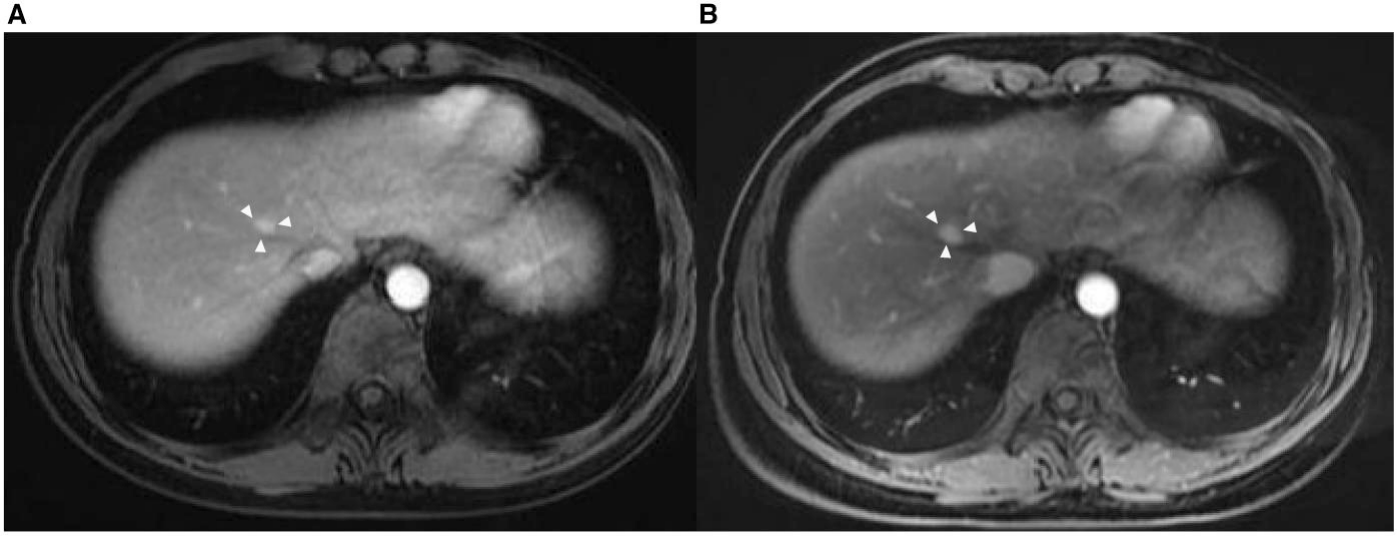
Liver metastasis during follow-up. (A) MRI image at 34 months postsurgery showing a small liver metastasis (white arrowheads). (B) Follow-up MRI at 38 months showing enlargement of the lesion prior to intervention with radiofrequency ablation and combination chemotherapy (white arrowheads). Abbreviation: MRI, magnetic resonance imaging.

Postoperative follow-up consisted of quarterly endocrine testing and alternating CT/magnetic resonance imaging every 6 months. At 34 months, a 4-mm segment VIII liver nodule emerged and enlarged to 12 mm over 4 months (Fig. 4A and 4B). In view of the small size, subcapsular location, and high Ki-67 index of the primary tumor, percutaneous radiofrequency ablation (RFA) was selected instead of hepatic resection [18]. Mitotane, previously tapered to maintenance, was re-escalated (1.0 → 1.5 g/day) and combined with 3 cycles of etoposide, doxorubicin, and cisplatin. Hydrocortisone and fludrocortisone were titrated.

**Figure 5.**
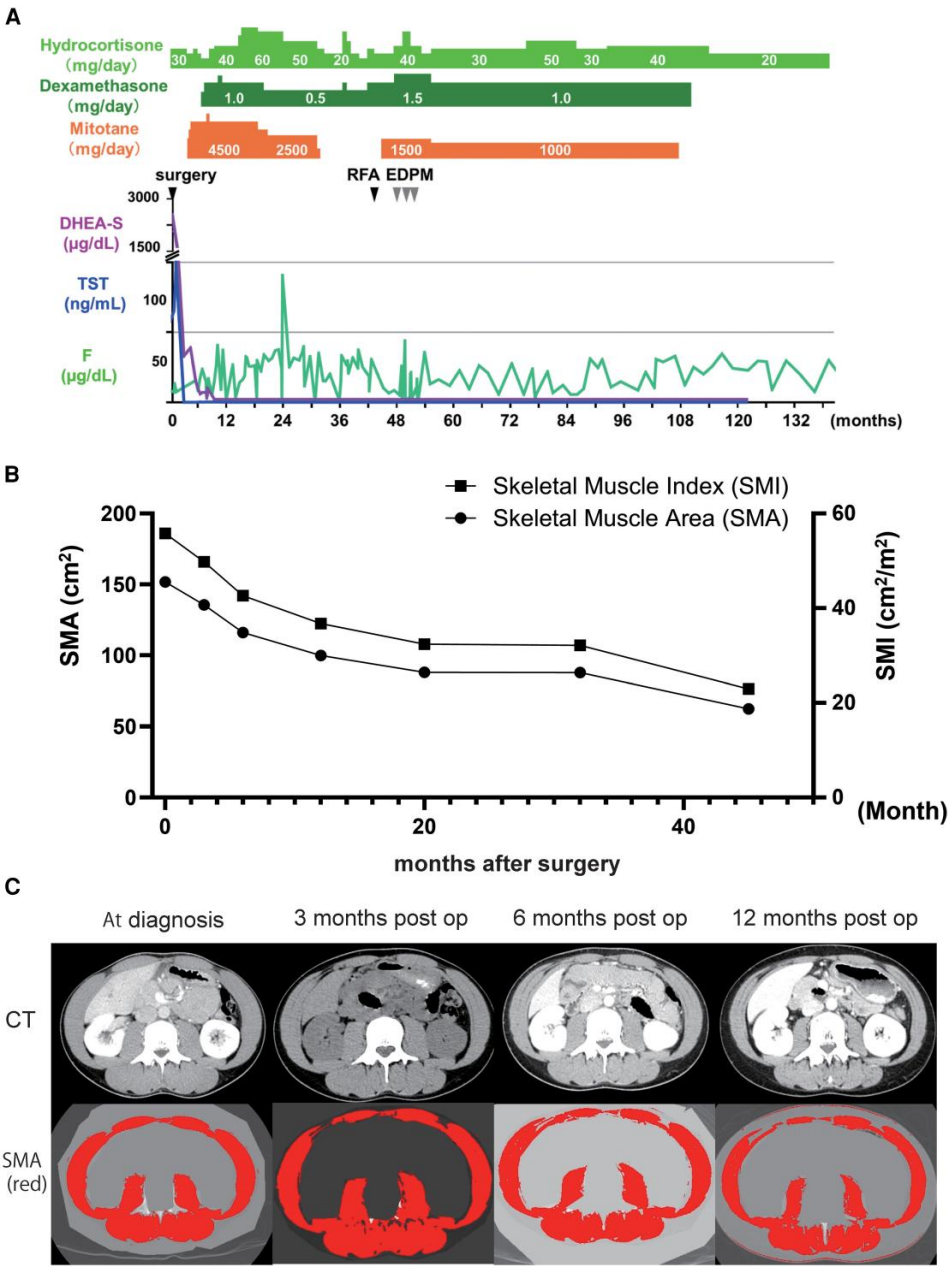
Comprehensive treatment course, hormonal profile, and body composition changes over 132 months. (A) Timeline showing therapeutic interventions and hormonal measurements during follow-up. Upper section displays hydrocortisone (light green), dexamethasone (dark green), and mitotane (orange) dosing. Lower section shows serum levels of dehydroepiandrosterone sulfate (purple), testosterone (blue), and cortisol (green, F). Black arrowheads indicate timing of surgery; radiofrequency ablation; and etoposide, doxorubicin, cisplatin, and mitotane chemotherapy. Note the rapid normalization of androgen levels following tumor resection and sustained normal levels throughout the follow-up period. (B) Progressive decline in skeletal muscle area (circles) and skeletal muscle index (squares) over 45 months following surgical resection. Both parameters show continuous reduction from pathologically elevated preoperative values to sarcopenic levels. (C) Representative axial computed tomography images at the L3 vertebral level demonstrating marked reduction in skeletal muscle mass over time. Upper row (left to right): preoperative, 3 months, 6 months postoperative. Lower row (left to right): 12 months, 20 months, 32 months postoperative. Note the progressive decrease in muscle area and increased intramuscular fat infiltration.

RFA achieved complete local control, and subsequent imaging has shown no recurrence for more than 8 years. Mitotane was discontinued after 2 consecutive years of negative scans while low-dose hydrocortisone (20 mg) was continued. Serum testosterone, DHEA-S, and androstenedione remain within reference limits (Fig. 5A).

Serial cross-sectional imaging at the L3 vertebral level quantified the reversal of androgen-driven hypertrophy [19]. Preoperative skeletal-muscle area and skeletal-muscle index (SMI) were 150.2 cm² and 55.8 cm²/m², respectively—well above female norms. Skeletal-muscle area/SMI fell to 120.1 cm² and 44.2 cm²/m² at 6 months, 95.2/35.0 cm²/m² at 20 months, and 62.5/23.0 cm²/m² at 45 months, representing an overall 59% decline. The final SMI lies below consensus sarcopenia thresholds for both sexes, corroborating the patient's subjective loss of explosive strength during daily activity. (Fig. 5B and 5C).

The patient’s menstrual cycles resumed 14 months after mitotane withdrawal and stabilized at 30- to 45-day intervals, indicating partial recovery of hypothalamic-pituitary-ovarian function. Formal postoperative athletic testing was not performed, but the patient noted greater fatigue in sprinting and throwing activities than during endurance exercise, consistent with the preferential androgen effect on fast-twitch type II fibers. Her body composition normalized with reduced shoulder and upper-arm definition and increased iliofemoral adiposity.

Co-expression of CYP17A1 and cytochrome *b*_5_ is crucial: cytochrome *b*_5_ enhances the 17,20-lyase reaction, diverting pregnenolone and progesterone toward androgen synthesis. This cooperative mechanism, documented in fewer than 10% of ACCs, explains the tumor's testosterone output. Weak CYP21A2 further restrains cortisol biogenesis, preventing adrenal insufficiency while permitting androgen precursors to accumulate. Minimal SULT2A1 hampers sulfation and clearance of DHEA. This biochemical milieu yielded a serum DHEA-S of 1970 µg/dL, among the highest in adolescent ACC, underscoring the diagnostic value of enzyme-level mapping.

This case illustrates that aggressive multimodal treatment—R0 adrenalectomy, sustained mitotane exposure, and definitive therapy for oligometastatic relapse—can deliver durable remission in high-risk adolescent ACC. Integration of immunohistochemical, genomic, and functional readouts clarified tumor biology; guided surveillance intensity; and provided objective documentation of the reversible anabolic impact of pathological hyperandrogenism. Ongoing management focuses on lifelong endocrine follow-up; bone-mineral monitoring; and counseling regarding fertility, pregnancy, and adrenal-crisis prevention. At the latest review—9 years after the initial operation—the patient remains recurrence-free, normotensive, and engaged in full-time study.

## Discussion

We report ACC-driven hyperandrogenism in a 17-year-old softball player achieving remission through surgery, mitotane, RFA, and chemotherapy. Key findings include (1) marked fiber-type-specific athletic performance gains from pathological androgens, (2) rare *BCOR*/*HDAC9* mutations expanding the molecular spectrum of ACC, and (3) aggressive treatment and long-term management yielding >8-year disease-free survival.

Supraphysiologic testosterone produced gains most evident in power tasks relying on fast-twitch (type II) fibers: grip strength almost doubled, and standing long-jump rose 46%. Endurance capacity, dependent on type I fibers, increased 26%. This differential mirrors laboratory work showing that androgens preferentially expand type II myofibers and increase motor-unit firing [20, 21], while oxidative capacity rises modestly. While a causal relationship is likely, underlying genetic or training-related factors may have contributed to the observed gains. In competitive sports, performance gains exceeding 2 SDs above population norms may trigger anti-doping investigations. Therefore, clinicians and regulatory authorities should consider underlying endocrine disorders, including rare tumors like ACC, to prevent diagnostic errors and avoid unjust sanctions [22].

Immunohistochemistry confirmed a steroidogenic profile diverting precursors toward testosterone: intense CYP17A1 + cytochrome *b*_5_, weak–moderate CYP21A2, strong HSD3B2, and minimal SULT2A1.

To better understand the molecular drivers of the tumor's aggressive and hyperandrogenic phenotype, we performed whole-exome sequencing, which uncovered 2 rare somatic mutations—*BCOR* P1384R and *HDAC9* R947P—in the absence of *TP53* or *CTNNB1* alterations. Each variant occurs in <3% of catalogued ACCs and is predicted pathogenic, expanding the spectrum of epigenetic regulators implicated in pediatric ACC. While the prognostic significance of these *BCOR/HDAC9* variants remains uncertain, their identification highlights the molecular heterogeneity of ACC beyond common *TP53/CTNNB1* alterations.

Although laparoscopic resection of tumors >6 cm remains controversial, we achieved R0 margins despite the 8-cm size, as imaging showed a well-encapsulated lesion without vascular invasion, our center had extensive laparoscopic experience, and the patient preferred a minimally invasive approach. Current guidelines for ACC management emphasize the importance of complete surgical resection and adjuvant therapy in high-risk cases [23]. Mitotane was titrated to 14 to 20 mg/L, and quarterly laboratory review plus semi-annual CT/magnetic resonance imaging provided surveillance. At 34 months, a segment VIII liver nodule emerged (4 mm) and grew to 12 mm within 4 months. Given its subcapsular location, percutaneous RFA was chosen [18]; mitotane was re-escalated and 3 cycles of etoposide, doxorubicin, and cisplatin added. This strategy achieved complete remission exceeding 8 years, far surpassing the ∼35% 5-year survival typical of stage II ACC. Long-term follow-up has focused on managing adrenal insufficiency, monitoring anthracycline cardiotoxicity risk, and addressing skeletal-muscle loss following androgen normalization.

Serial L3 CT images showed an SMI decline from 55.8 to 23.0 cm²/m² (−59%) over 45 months—below the 29 cm²/m² threshold for young women—mirroring loss of explosive power. Menstruation resumed 14 months after mitotane cessation, and body composition shifted toward a typical female pattern, highlighting the reversible—but quality-of-life-relevant—impact of pathological androgen excess. Given the substantial quality-of-life impact of androgen withdrawal and treatment sequelae, survivorship planning for young women with ACC must also include fertility preservation, psychosocial support, and adrenal-crisis preparedness.

Durable remission also demands structured toxicity monitoring. Mitotane can impair cognition and raise lipids, while cumulative anthracycline doses threaten cardiomyopathy. Our protocol includes annual echocardiography, lipid profiling, neurocognitive testing, dual-energy X-ray absorptiometry scans, and emergency hydrocortisone education cards.

Study limitations include uncertain prognostic significance of BCOR/HDAC9 variants and single-case design. Multicenter registries are needed to validate these findings and refine ACC risk stratification in athletes.

## Learning Points

- Pathological hyperandrogenism from ACC can dramatically enhance athletic performance, with reversible effects following treatment.
- Aggressive multimodal therapy (surgery, mitotane, RFA for oligometastatic disease) achieved long-term remission in this high-risk ACC (Ki-67 14%), suggesting potential benefit of comprehensive treatment in young patients.
- Exceptional athletic gains with virilization in young athletes warrant endocrine evaluation to exclude underlying pathologies.
- Young women with ACC require comprehensive care addressing both oncological outcomes and quality-of-life issues, including fertility and adrenal insufficiency.
- Clinicians and sports authorities must remain vigilant for pathological hyperandrogenism, which can mimic doping and lead to misclassification in athletic contexts.

## Data Availability

Some or all datasets generated during and/or analyzed during the current study are not publicly available but are available from the corresponding author on reasonable request.

## References

[1] Sharma E, Dahal S, Sharma P, et al The characteristics and trends in adrenocortical carcinoma: a United States population-based study. J Clin Med Res. 2018;10(8):636‐640. 29977421 10.14740/jocmr3503w PMC6031252

[2] Else T, Kim AC, Sabolch A, et al Adrenocortical carcinoma. Endocr Rev. 2014;35(2):282‐326. 24423978 10.1210/er.2013-1029 PMC3963263

[3] Kadi F . Cellular and molecular mechanisms responsible for the action of testosterone on human skeletal muscle. A basis for illegal performance enhancement. Br J Pharmacol. 2008;154(3):522‐528. 18414389 10.1038/bjp.2008.118 PMC2439525

[4] Bhasin S, Woodhouse L, Casaburi R, et al Testosterone dose-response relationships in healthy young men. Am J Physiol Endocrinol Metab. 2001;281(6):E1172‐E1181. 11701431 10.1152/ajpendo.2001.281.6.E1172

[5] Jiang C, Yin L, Pu Q, Ke C. Giant androgen-producing adrenocortical carcinoma causing male characteristics. Asian J Surg. 2024;47(1):718‐720. 37903692 10.1016/j.asjsur.2023.10.005

[6] Bansode RD, Singh SK. A study of muscular endurance and strength ability of softball players. IJIREEICE. 2022;10:121‐124.

[7] de Almeida-Neto PF, de Matos DG, Pinto VCM, et al Can the neuromuscular performance of young athletes be influenced by hormone levels and different stages of puberty? Int J Environ Res Public Health. 2020;17(16):5637. 32764284 10.3390/ijerph17165637 PMC7460253

[8] Terzolo M, Zaggia B, Allasino B, De Francia S. Practical treatment using mitotane for adrenocortical carcinoma. Curr Opin Endocrinol Diabetes Obes. 2014;21(3):159‐165. 24732405 10.1097/MED.0000000000000056

[9] Terzolo M, Daffara F, Ardito A, et al Management of adrenal cancer: a 2013 update. J Endocrinol Invest. 2014;37(3):207‐217. 24458831 10.1007/s40618-013-0049-2

[10] Berruti A, Baudin E, Gelderblom H, et al Adrenal cancer: ESMO clinical practice guidelines for diagnosis, treatment and follow-up. Ann Oncol. 2012;23(Suppl 7):vii131‐vii138. 22997446 10.1093/annonc/mds231

[11] Reimondo G, Puglisi S, Zaggia B, et al Effects of mitotane on the hypothalamic-pituitary-adrenal axis in patients with adrenocortical carcinoma. Eur J Endocrinol. 2017;177(4):361‐367. 28780517 10.1530/EJE-17-0452

[12] Bedrose S, Daher M, Altameemi L, Habra MA. Adjuvant therapy in adrenocortical carcinoma: reflections and future directions. Cancers (Basel). 2020;12(2):508. 32098326 10.3390/cancers12020508 PMC7072549

[13] Sportoletti P, Sorcini D, Falini B. BCOR gene alterations in hematologic diseases. Blood. 2021;138(24):2455‐2468. 33945606 10.1182/blood.2021010958 PMC8887995

[14] Xu W, Wu H, Shen X, et al The role of HDAC family in human cancer and its potential use in diagnosis and prognosis. Front Oncol. 2022;12:889478.

[15] Assié G, Letouzé E, Fassnacht M, et al Integrated genomic characterization of adrenocortical carcinoma. Nat Genet. 2014;46(6):607‐612. 24747642 10.1038/ng.2953

[16] Crona J, Beuschlein F. Adrenocortical carcinoma—towards genomics guided clinical care. Nat Rev Endocrinol. 2019;15(9):548‐560. 31147626 10.1038/s41574-019-0221-7

[17] Zheng S, Cherniack AD, Dewal N, et al Comprehensive pan-genomic characterization of adrenocortical carcinoma. Cancer Cell. 2016;29(5):723‐736. 27165744 10.1016/j.ccell.2016.04.002 PMC4864952

[18] Wood BJ, Abraham J, Hvizda JL, Alexander HR, Fojo T. Radiofrequency ablation of adrenal tumors and adrenocortical carcinoma metastases. Cancer. 2003;97(3):554‐560. 12548596 10.1002/cncr.11084 PMC2443414

[19] Mourtzakis M, Prado CMM, Lieffers JR, Reiman T, McCargar LJ, Baracos VE. A practical and precise approach to quantification of body composition in cancer patients using computed tomography images acquired during routine care. Appl Physiol Nutr Metab. 2008;33(5):997‐1006. 18923576 10.1139/H08-075

[20] Bhasin S, Taylor WE, Singh R, et al The mechanisms of androgen effects on body composition: mesenchymal pluripotent cell as the target of androgen action. J Gerontol A Biol Sci Med Sci. 2003;58(12):1103‐1110. 10.1093/gerona/58.12.m1103 14684707

[21] Handelsman DJ, Hirschberg AL, Bermon S. Circulating testosterone as the hormonal basis of sex differences in athletic performance. Endocr Rev. 2018;39(5):803‐829. 30010735 10.1210/er.2018-00020 PMC6391653

[22] Casto KV, Arthur LC, Hamilton DK, Edwards DA. Testosterone, athletic context, oral contraceptive use, and competitive persistence in women. Adapt Human Behav Physiol. 2022;8(1):52‐78.

[23] Fassnacht M, Dekkers OM, Else T, et al European Society of Endocrinology Clinical Practice Guidelines on the management of adrenocortical carcinoma in adults, in collaboration with the European Network for the Study of Adrenal Tumors. Eur J Endocrinol. 2018;179(4):G1‐G46. 30299884 10.1530/EJE-18-0608

